# Maternal-fetal complications of non-immune fetal hydrops – mirror syndrome and hypereactio luteinalis with ovarian torsion: A case report

**DOI:** 10.1016/j.crwh.2025.e00775

**Published:** 2025-12-12

**Authors:** Helena C. Bartels, Greg Ryan, Edgar Jaeggi, Homero Flores-Mendoza

**Affiliations:** Department of Obstetrics and Gynecology, Ontario Fetal Centre, Mount Sinai Hospital, University of Toronto, Toronto, ON, Canada

**Keywords:** Mirror syndrome, Fetal hydrops, Hypereactio luteinalis, Preeclampsia

## Abstract

Mirror syndrome, previously referred to as Ballantyne's syndrome, is a rare obstetric disorder characterized by maternal edema in association with fetal hydrops and placental edema. This report concerns the case of a 35-year-old multiparous woman who developed mirror syndrome secondary to fetal supraventricular tachycardia complicated by hydrops and intrauterine fetal demise. Her pregnancy was further complicated by hypereactio luteinalis with ovarian torsion requiring surgical detorsion. The case highlights the challenges of managing maternal and fetal hydrps, and its rare association with hypereactio luteinalis.

## Highlights

• This report concerns a rare case of fetal arrhythmia leading to fetal and maternal hydrops.
• The patient subsequently developed maternal mirror syndrome.
• Unusual association with ovarian hypereactio luteinalis, resulting in ovarian torsion.
• The case highlights the rare association of HL with fetal and maternal hydrops, and the need for clinical vigilance over the potential to develop severe and acute mirror syndrome.

## 1 Introduction

Mirror syndrome, first described by Ballantyne in 1892, is characterized by the triad of maternal oedema, placental oedema, and fetal hydrops [1]. However, the diagnostic criteria used to define mirror syndrome varies widely between studies, with a recent systematic review highlighting the lack of consensus and significant heterogeneity in the literature [2]. The pathogenesis of mirror syndrome remains incompletely understood; however, it is reported to involve placental injury with trophoblastic damage, resulting in widespread maternal endothelial dysfunction as a result of release of placental-derived anti-angiogenic factors such as sFLT-1 and inflammatory cytokines [3,4]. As a result of excessive sFLT-1 secretion, vascular endothelial growth factor and placental growth factor (PLGF) are suppressed in mirror syndrome, leading to endothelial dysfunction and the systemic sequalae seen in mirror syndrome [5]. These mechanisms are similar to the pathways resulting in preeclampsia [4,6].

Mirror syndrome is estimated to complicate 5–30 % of fetal hydrops cases. The maternal presentation has several overlapping clinical features with preeclampsia – such as oedema and hypertension; however, mirror syndrome typically shows haemodilution rather than haemoconcentration [3,4,7]. Mirror syndrome can involve a varied clinical picture, which may include hypertension, pulmonary oedema, acute kidney injury and, rarely, cardiac failure [2,8]. Fetal outcomes are poor, with a live birth rate of less than 10 % reported in several studies [2,9].

Hypereactio luteinalis (HL) is a benign, rare ovarian condition characterized by bilateral multicystic ovarian enlargement, likely linked to elevated β-hCG [10]. Its association with mirror syndrome is very rare, hence this case has several important learning points for clinicians.

## 2 Case Presentation

A 35-year-old woman (G4P3) with a BMI of 33 m/kg^2^ presented with a spontaneous, planned pregnancy. Her relevant medical history included polycystic ovarian syndrome. Early first-trimester screening revealed an increased nuchal translucency (4.2 mm) with high-risk combined screening for trisomy 21 (1:13). However, non-invasive prenatal testing returned a low-risk result, and further testing with amniocentesis was declined. A fetal echocardiograph at 18 weeks found a normal cardiac structure and function.

At 21 + 1 weeks, ultrasound demonstrated fetal hydrops with ascites, pleural effusion, scalp oedema, and placentomegaly. The fetus was noted to have a sustained tachycardia of 260 beats per minute. Fetal cardiology confirmed 1:1 atrioventricular re-entrant tachycardia (AVRT) at 240 bpm with associated hydrops. The patient was admitted for inpatient observation and commenced on maternal flecainide and digoxin for transplacental antiarrhythmic therapy. Following treatment, conversion to normal sinus rhythm with 1:1 conduction was achieved at 22 + 2 weeks.

At reassessment at 23 + 2 weeks, the fetus was in normal sinus rhythm, but remained hydropic with persistent ascites, pleural effusion and scalp oedema. At this time, maternal ultrasound revealed massively enlarged bilateral ovaries with large theca lutein cysts consistent with hypereactio luteinalis. The ovaries had previously been normal. The left ovary measured 13 × 8 × 10 cm with cysts up to 9 × 2.5 cm, and the right ovary measured 8.7 × 5.6 × 9 cm. Serum levels of FSH (<0.1 IU/L), LH (1.4 IU/L), prolactin (366 μg/L) and estradiol (>55,000 pmol/L) were all found to be altered consistent with pregnancy changes. The patient experienced abdominal discomfort, which resolved with analgesia, consistent with the ovarian enlargement. Blood pressure remained normal at 118/83 mmHg.

At 25 weeks, the patient presented with reduced fetal movements. Intrauterine fetal demise was diagnosed. She was hypertensive at 160/90 mmHg, with maternal serum significant for a haemoglobin of 101 g/dL, platelets of 181 cells/dL, a mildly raised creatinine of 75 μmol/L, and mild proteinuria (protein creatine ratio 70). Placental growth factor (PIFG) was 59 pg/mL. During induction with misoprostol, she developed dyspnoea and chest tightness, with a chest X-ray confirming pulmonary oedema. Oxygen saturation dropped to 88–90 %, requiring supplemental oxygen, while rising blood pressure required increasing doses of labetalol and nifedipine throughout the labour induction. Pulmonary oedema was managed with diuretic therapy. The patient also had worsening kidney function with a marked elevation in creatinine peaking at 145 umol/L. Magnesium sulphate was administered for severe preeclampsia/mirror syndrome. She delivered a stillborn hydropic fetus vaginally the following day. Postpartum recovery was rapid, with normalization of renal function, oxygen saturation, and resolution of maternal oedema within 72 h postpartum. She was discharged home well on day 2. Given the enlarged ovaries bilaterally, which were still present on day 2 postpartum, she was advised of the possible risk of complications including ovarian torsion and a follow-up within two weeks was planned.

Placental histopathology was significant for placentomegaly with a placental weight greater than the 95th percentile for gestation, with widespread villous hydrops, marked excess of circulating nucleated haematopoietic elements and fetal vascular malperfusion.

Two weeks postpartum, the patient re-presented with severe left flank and lower abdominal pain, requiring opiate analgesia. Clinically the findings were consistent with ovarian torsion, which was confirmed on ultrasound, which was also significant for absent blood flow to the left ovarian pedicle. Laparoscopy found a left ovarian torsion with three twists on the ovarian pedicle, requiring detorsion and bilateral ovarian cyst drainage (11 cysts on the left, 3 cysts on the right). Histopathology confirmed hypereactio luteinalis. The patient recovered well postoperatively.

## 3 Discussion

This case presents a rare association of non-immune fetal hydrops and subsequent maternal mirror syndrome and HL complicated by ovarian torsion. It offers several important clinical lessons. First, careful monitoring for mirror syndrome should be considered in patients with evidence of fetal hydrops who begin to develop symptoms such as hypertension and maternal oedema. As demonstrated, mirror syndrome can develop rapidly with sudden maternal deterioration, hence close monitoring for signs such as hypertension and edema is important.

Second, in this case, fetal hydrops was attributable to supraventricular tachycardia (SVT), a recognized but uncommon aetiology. Fetal arrhythmias occur in approximately 1 in 4000 live births, with sustained SVT being a well-established cause of non-immune hydrops [11]. Current evidence supports the use of flecainide and sotalol as first-line agents for rhythm control in hydropic fetuses, with superiority over digoxin due to improved placental transfer and higher conversion rates [11]. Despite appropriate therapy, resolution of hydrops does not always follow, and the prognosis is poor once hydrops has become established. In this case, fetal demise occurred despite rhythm control and intensive monitoring, underscoring the poor fetal outcomes reported in association with fetal hydrops and maternal mirror syndrome. Hence it is important to counsel parents of the possible poor prognosis even in cases which may be amenable to therapy.

Third, the presence of HL provided an additional management challenge in this case. HL is rare and typically benign, associated with elevated hCG levels in molar pregnancies, multiple gestations, and occasionally hydrops [10,12,13]. In this patient, HL was complicated by torsion, requiring surgical intervention. Usually, spontaneous postpartum resolution is expected but, as demonstrated by the present case, vigilance for acute complications such as torsion is important even after delivery. To the authors' knowledge, there are fewer than five cases of non-immune fetal hydrops associated with HL reported in the literature, with only one published within the previous 30 years [13,14]. In the present case, the placenta was extremely enlarged and oedematous. This supports the theory that the multi-cystic ovaries were secondary to increased secretion of placental hormones such as beta-HCG [13]. This is further supported by a previous case report which found normal levels of pituitary hormones such as follicular stimulating hormone, prolactin and growth hormone in both maternal serum and the cysts' aspirate [14]. In the present case, maternal serum hormonal levels were consistent with expected ranges for pregnancy; however, their value in the clinical context is likely limited given their uncertain interpretability in pregnancy.

Finally, it is important to note the variable maternal outcomes in mirror syndrome [2]. Complications such as pulmonary oedema, acute kidney injury, and hypertensive disease are well documented, with severe maternal complications occurring in up to 90 % of cases [2,9]. However, unlike preeclampsia, where delivery is needed to treat the maternal disease, in cases of fetal hydrops which are amenable to fetal therapy, fetal and maternal outcomes can be more favourable and delivery is not necessitated. Where fetal therapy is successful, maternal symptoms often completely resolve while live birth rates of above 80 % are reported in some studies [7,15].

## 4 Conclusion

Mirror syndrome remains a rare but serious obstetric complication with high maternal and fetal morbidity. This case highlights the rare co-existence of HL, which here was complicated by torsion, with fetal and maternal hydrops. Early recognition, multidisciplinary management, and postpartum surveillance are essential for optimising outcomes.

## Contributors

Helena C. Bartels contributed to patient care, conception of the case report, acquiring and interpreting the data, drafting the manuscript and undertaking the literature review.

Greg Ryan contributed to patient care, conception of the case report, acquiring and interpreting the data and drafting the manuscript.

Edgar Jaeggi contributed to patient care, acquiring and interpreting the data and revising the article critically for important intellectual content.

Homero Flores-Mendoza contributed to patient care, drafting the manuscript and revising the article critically for important intellectual content.

All authors approved the final submitted manuscript.

## Patient consent

Written informed consent was obtained from the patient for publication of the case report.

## Provenance and peer review

This article was not commissioned and was peer reviewed. Helena Bartels, an editor of *Case Reports in Women's Health*, was not involved in editorial consideration of the manuscript and was blinded to the process.

## Funding

This work did not receive any specific grant from funding agencies in the public, commercial, or not-for-profit sectors.

## Declaration of competing interest

The authors declare that they have no competing interest regarding the publication of this case report.
